# Adverse fetal/neonatal and obstetric outcomes in pregnant women with heart disease: Data from the Registry of Pregnancy and Cardiac Disease (ROPAC) of the Kermanshah, Iran

**DOI:** 10.1371/journal.pone.0352657

**Published:** 2026-07-17

**Authors:** Nasrin Mansouri, Ali Azizi, Maryam Sayyadi, Marzieh Bagherinia

**Affiliations:** 1 Department of Obstetrics and Gynecology, Clinical Research Development Center, Imam Reza Hospital, Kermanshah University of Medical Sciences, Kermanshah, Iran; 2 Department of Community and Family Medicine, School of Medicine, Kermanshah University of Medical Sciences, Kermanshah, Iran; 3 Clinical Research Development Center, Imam Reza Hospital, Kermanshah University of Medical Sciences, Kermanshah, Iran; 4 PhD of Reproductive Health, Clinical Research Development Center, Motazedi Hospital, Kermanshah University of Medical Sciences, Kermanshah, Iran; Xiangya Hospital Central South University, CHINA

## Abstract

**Background:**

Pregnancy complicated by heart disease can pose serious risks and complications for both the mother and the fetus. Understanding the prevalence of these conditions during pregnancy, along with the various types of cardiac diseases and their associated complications, is essential for reducing maternal and fetal morbidity and mortality during pregnancy and childbirth.

**Objective:**

This study aims to examine the clinical characteristics, risk factors, and outcomes of heart disease in pregnant women.

**Methods:**

This single-center prospective registry-based cohort study without a comparison group (single arm) was conducted using data from the Cardiac Disease Registry for pregnant women in Kermanshah, Iran. All pregnant women with structural heart diseases-including congenital heart disease, valvular disease, prosthetic heart valves, cardiomyopathy, ischemic heart disease, aortopathy, and arrhythmias-were included. Data were collected using validated checklists and entered into an online registry.

**Results:**

A total of 36 pregnant women with heart disease were included. The most common diagnoses were congenital heart disease (38.9%), valvular heart disease (30.6%), and cardiomyopathy (22.2%). Most patients (94.5%) had preserved left ventricular ejection fraction (LVEF ≥40%), and 88.9% had pre-existing cardiac disease, with 86.2% receiving pre-pregnancy cardiac counseling. No cases of maternal or neonatal mortality were observed. New-onset heart failure occurred in 13.9% of women, and 27.8% required hospitalization for cardiac reasons during pregnancy. The total abortion rate was 19.4% (11.1% spontaneous, 8.3% therapeutic). Preterm birth occurred in 24.1% of cases, and low birth weight in 17.2%. The cesarean delivery rate was high (80.6%). Hypertensive disorders and gestational diabetes were relatively uncommon. No congenital heart defects were observed in neonates.

**Conclusion:**

Comprehensive pre-pregnancy counseling, specialized prenatal care, and multidisciplinary management can lead to favorable maternal and neonatal outcomes in high-risk pregnancies complicated by heart disease. The absence of mortality in this cohort highlights the effectiveness of organized care models. Despite the limited sample size, these findings underscore the value of structured, registry-based approaches to improving outcomes in pregnant women with cardiac disease.

## Introduction

Pregnancy imposes substantial physiological stress on the cardiovascular system, increasing the risk of heart failure, arrhythmias, thrombosis, and aortic dissection during gestation and the early postpartum period [1]. The prevalence of pregnancy-related heart disease has risen to 1–4%, driven by factors such as advanced maternal age, unhealthy lifestyles, and improved diagnostic capabilities [2]. Consequently, cardiovascular disease has become the leading cause of maternal mortality in developed countries, with women who have pre-existing or congenital heart conditions being particularly vulnerable [3,4].

Women with heart disease face significantly elevated risks of adverse maternal and fetal outcomes, including heart failure, arrhythmias, thromboembolism, preterm birth, intrauterine growth restriction, and stillbirth [5–7]. Despite these well-documented risks, the management of cardiac conditions during pregnancy remains complex. Hemodynamic changes can exacerbate existing disease, while fetal safety concerns limit diagnostic and therapeutic options [8]. Furthermore, the lack of robust evidence from randomized controlled trials in pregnant populations forces clinicians to rely heavily on clinical experience and expert consensus [9]. Risk factors associated with these adverse outcomes include advanced maternal age, smoking, multiparity, anticoagulant therapy, poor cardiac function, cyanosis, and left heart obstruction [10–12]. Furthermore, the impact of cardiac disease extends beyond traditional clinical endpoints; it can also negatively affect the mother’s subjective experience of childbirth and her functional recovery postpartum [13]. A systematic review highlights that a significant proportion of women experience a negative birth experience [14], while other research indicates that physical activity levels during pregnancy are closely linked to the functional status of mothers after delivery [15]. However, as a result of recent progress in cardiology and cardiac surgery, the lifespan of patients with congenital heart disease has been prolonged and therefore the number of pregnant women with such conditions has gone up.

Early detection and proper treatment of heart disease during pregnancy can be instrumental in lessening the risk of disease and death linked to it. Through the systematic registration and analysis of heart disease cases in pregnancy, it is possible to gain deeper insights of clinical patterns, risk factors, and outcomes which in turn leads to better management strategies. This research is to find out the clinical features, risk factors, and outcomes of heart disease among pregnant women through the gathering and analysis of data from 36 documented cases over three years. The results are expected to lead to better clinical protocols, enhanced maternal and neonatal outcomes, and be a platform for future research in this area.

## Methods

### Study design

This was a single-center prospective observational cohort study conducted at Imam Reza Hospital, a Tertiary Referral Center for Cardiac Diseases during Pregnancy in Kermanshah, Iran. Data were collected prospectively through the Heart Disease in Pregnancy Registry of Kermanshah University of Medical Sciences. The registry, which was set up by the Disease Registry Unit, comprises pregnant women diagnosed with structural heart diseases such as congenital heart disease, valvular heart disease, prosthetic heart valves, cardiomyopathy, ischemic heart disease, aortopathy, pulmonary arterial hypertension, and arrhythmias. These patients were recruited from April 2022 to March 2025.

The final diagnoses were made based on clinical history, medical records, and echocardiographic findings. Experienced cardiologists performed echocardiography with standard instruments and it included the assessment of cardiac chamber sizes, left ventricular ejection fraction (LVEF), valvular function (stenosis and regurgitation), pulmonary artery pressure, and the presence of intracardiac shunts. All measurements were performed in accordance with the American Society of Echocardiography (ASE) guidelines for chamber quantification (REF), valvular disease evaluation (REF), and right heart assessment (REF) [16].

### Data collection

All eligible pregnant women with structural heart disease requiring higher-level medical care were consecutively enrolled at the specialized tertiary referral centre for cardiac diseases in pregnancy — Imam Reza Hospital. Through the Heart Disease in Pregnancy Registry, participants were identified either as those with pre-existing cardiac diagnoses who had received pre-pregnancy counseling and ongoing follow-up, or as those newly diagnosed during pregnancy. A multidisciplinary team consisting of cardiologists, obstetricians, and maternal–fetal medicine specialists managed all patients according to their individual clinical needs.

After the diagnostic confirmation, information about the patients was first captured on researcher-made paper forms and later a designated data manager would upload the information weekly into the centralized online database (https://rabit.ir/registry/). The physician in charge was responsible for the confirmation of all the data entries. The data collection checklist which was made after the exhaustively reviewed literature included: demographic information (age, education, occupation, self-reported income, body mass index, pregnancy history); clinical information (type of heart disease, history of cardiac interventions, medications before and during pregnancy), cardiovascular risk factors and comorbidities; pregnancy outcomes (maternal and fetal complications). All the participants signed the informed consent and patient information was kept anonymous by using coded identifiers. The checklist’s accuracy was ensured before the study commencement through ten different specialists in obstetrics and cardiology, who reviewed the checklist and provided their feedback and changes were integrated accordingly.

Once the patients had been registered, they were kept under regular observation during the whole period of their pregnancy and they could be seen either by phone or in-person every 4–6 weeks, according to the clinical status and the doctor’s judgement. Any complications or hospitalizations were promptly conveyed to the registry staff. Delivery counseling for all patients was at Imam Reza Hospital and postpartum follow-up was up to 18 months long, with monthly phone or in-person appointments to evaluate maternal and neonatal outcomes.

Pregnancy and delivery outcomes consisted of a broad variety of complications of the mother and the fetus, which were identified according to globally recognized standards (WHO and ACOG). The following maternal outcomes were evaluated: spontaneous abortion, therapeutic abortion, preterm birth (delivery before 37 completed weeks of gestation), intrauterine growth restriction (IUGR), preeclampsia or HELLP syndrome, gestational diabetes mellitus (GDM) or glucose intolerance first diagnosed between 24 and 28 weeks, mode of delivery (vaginal or cesarean section, divided into cardiac or obstetric indication), postpartum hemorrhage (blood loss ≥500 mL for vaginal delivery or ≥1000 mL for cesarean section), maternal mortality (up to 42 days postpartum), and severe maternal morbidity as per WHO criteria. Fetal and neonatal outcomes were intrauterine fetal demise (IUFD), low birth weight (<2500 g), small for gestational age (birth weight <10th percentile), Apgar score <7 at 5 minutes, preterm delivery, neonatal death (within 28 days of life), and congenital heart defects diagnosed by neonatal echocardiography [17–20].

All live-born neonates underwent systematic cardiac evaluation. This included a comprehensive physical examination by a pediatrician within 24 hours of birth, with particular attention to cardiac auscultation, peripheral pulse assessment, and oxygen saturation monitoring. Neonates with any abnormal findings (e.g., pathological murmurs, cyanosis, tachypnea, or oxygen saturation <95% in room air) were referred for transthoracic echocardiography performed by a pediatric cardiologist. Neonates who passed the initial screening and remained asymptomatic during the postnatal period were considered to have no congenital heart disease [21,22].

Based on the Clark risk classification for maternal cardiac mortality, patients were grouped. The Clark risk classification for maternal cardiac mortality is a tool that has been validated in many studies and is very often used for risk stratification in pregnant women with cardiac disease. Besides, it has a good predictive value for the occurrence of adverse cardiac events [23]. The classification system divides patients into the following categories:

Class I: Mild cardiac lesions without any hemodynamic compromise (e.g., small atrial septal defect, mild valvular regurgitation); maternal risk is negligible.
Class II: Moderate cardiac disease (e.g., moderate mitral stenosis); associated with a low to moderate maternal risk (approximately 5–15%).
Class III: Severe structural or functional cardiac disease with significant limitation of activity (e.g., severe valvular stenosis, cardiomyopathy with LVEF 30–40%); maternal risk is substantially increased (25–50%).
Class IV: Conditions associated with an extremely high maternal mortality risk (e.g., pulmonary arterial hypertension, severe cardiomyopathy with LVEF <30%); pregnancy is generally contraindicated in this group.

The Clark class of each patient was identified by a cardiologist based on the echocardiographic findings, hemodynamic parameters, and overall clinical status at the first prenatal evaluation.

### Ethical consideration

The study was approved by Kermanshah University of Medical Sciences, Kermanshah, Iran, with the ethical code IR.KUMS.REC.1397.856. All ethical principles, such as obtaining written informed consent from all participants, providing a comprehensive explanation by the researcher about the goals, methods, and reasons for conducting the study to the participants, the participants’ right to withdraw from the study at any stage of the study, and assuring the participants about the confidentiality of the information, were observed in this study.

### Statistical analysis

Data analysis was performed using SPSS version 26. Descriptive statistics, such as mean and standard deviation, were used to report quantitative data, and frequency and percentage were used for categorical data to describe demographic variables and pregnancy and delivery outcomes.

## Results

Table 1 shows the baseline characteristics of 36 pregnant women with heart disease who were the subjects of this study. The average maternal age was 33.8 years. More than half of the women (52.8%) had the history of two or more pregnancies, and their mean body mass index was 27.8 kg/m². As for the level of education, 25% were either illiterate or had only primary education, 30.5% had middle or high school education, 33.3% had a diploma, and 11.1% had a university degree. Most of the women (83.3%) were housewives. Speaking of income, 61.1% of the women described their income as severely inadequate, and 27.8% as inadequate. Importantly, 88.9% of the women had heart disease before the study, and among those with pre-pregnancy cardiac diagnoses, 86.2% had gone through cardiac counseling before conception. Moreover, 16.7% of the women had a history of chronic hypertension, and 5.5% had a history of diabetes. There was no smoking among the women in the study.

**Table 1:** Baseline characteristics of women with heart disease during pregnancy (n = 36).

Variables		Variables	
**Maternal age (year)**	33.8 ± 8.5	**Current smoker**	
**Gravidity**		Yes	0 (0%)
**1**	17 (47.2%)	No	36 (100%)
**=> 2**	19 (52.8%)	**Prior chronic hypertension**	
**Body mass index (BMI, kg/m**^**2**^)	27.8 ± 6.9	Yes	6 (16.7%)
**Gestational age at the time of study (weeks)**	8.6 ± 3.1	No	26 (72.2%)
**Education**		Unknown	4 (11.1%)
Illiterate/Elementary school	9 (25%)	**Prior diabetes mellitus**	
Guidance/ High school	11 (30.5%)	Yes	2 (5.5%)
Diploma	12 (33.3%)	No	32 (88.9%)
Academic	4 (11.1%)	Unknown	2 (5.5%)
**Job**		**Pre-pregnancy cardiac disease**	
Housewife	30 (83.3%)	Yes	32 (88.9%)
Working at home or outside the home	6 (16.7%)	No	4 (11.1%)
**Income**		**Pre-pregnancy Cardiac Counseling**	
Very insufficient	22 (61.1%)	Yes	31 (86.2%)
Insufficient	10 (27.8%)	No	1 (2.7%)
Somewhat sufficient	4 (11.1%)	Not Applicable*	4 (11.1%)

Data in n (%) unless otherwise specified.

*Not Applicable: applies to individuals without a pre-pregnancy cardiac diagnosis.

Table 2 Displays the clinical characteristics and medicine usage of pregnant women suffering from cardiac disease. The first three main causes of heart disease were congenital heart defects 38.9%), valvular heart diseases (30.6%), and cardiomyopathies (22.2%). Only 5.5% of the patients had aortopathy, and 2.8% had ischemic heart diseases. There were no patients with pulmonary hypertension. Most of them (94.5%) had estimated left ventricular ejection fraction (LVEF) of more than 40%. Based on Clark classification, patients in Class I accounted for 30.6%, Class II 36.1%, Class III 25.0%, and Class IV 8.3%. The majority of patients 88.9% were in sinus rhythm. Ventricular arrhythmia and sinus tachycardia were each 2.8% only. One-third of patients (34.4%) were receiving beta-blockers before pregnancy, and one in eight (12.5%) were receiving ACE inhibitors. During pregnancy, 5.6% of patients used diuretics. Heart failure symptoms were recorded in 13.9% of patients, and atrial fibrillation/flutter in 5.6%. These figures indicate that there were different kinds of cardiac diseases in this cohort, and hence the management of them during pregnancy had to be close and individualized.

**Table 2:** Clinical characteristics and medications used by pregnant women with heart disease.

Variables		Variables	
**Diagnosis details**		Yes	5 (13.9%)
Congenital heart disease	14 (38.9%)	No	31 (86.1%)
Valvular heart disease	11 (30.6%)	**Atrial fibrillation/flutter**	
Cardiomyopathy	8 (22.2%)	Yes	2 (5.6%)
Aortopathy	2 (5.5%)	No	34 (94.4%)
Ischaemic heart disease	1 (2.8%)	**Estimated LVEF <40%**	
Pulmonary hypertension	0 (0%)	Yes	2 (5.5%)
**Pre-pregnancy cardiac medication use**		No	34 (94.5%)
Diuretics	3 (9.4%)	***Clark Class***	
Beta-blockers	11 (34.4%)	I	11(30.6%)
ACE-inhibitors	4 (12.5%)	II	13(36.1%)
Other cardiac medication	13 (40.6%)	III	9(25.0%)
Anticoagulation	1 (3.1%)	IV	3(8.3%)
Not Applicable*	4 (11.1%)	***Heart rhythm (*on EKG)**	
**Diuretic use during pregnancy**		Sinus rhythm	32 (88.9%)
Yes	2 (5.6%)	Ventricular arrhythmia	1 (2.8%)
No	34 (94.4%)	Sinus tachycardia	1 (2.8%)
**Signs of heart failure**		Unknown	2 (5.5%)

Data in n (%) unless otherwise specified.

*Not Applicable: applies to individuals without a pre-pregnancy cardiac diagnosis.

The cardiac, obstetric, and neonatal outcomes of the 36 pregnant women with heart disease were summarized in Table 3.

**Table 3:** Pregnancy and delivery outcomes in women with heart disease during pregnancy (n = 36).

Variables		Variables	
**Maternal cardiac outcomes**		Intrauterine Fetal Demise (IUFD)	0 (0%)
Maternal mortality^a^	0 (0%)	Intrauterine-growth-retardation (IUGR)	1 (3.4%)
New- onsetheart failure	5 (13.9)	**Delivery**	
Hospital admission for cardiac reason during pregnancy	10 (27.8%)	Gestational age at delivery, mean ±SD	35.2 ± 2.4
Intraoperative Cardiac Complications during Cesarean Section	5 (17.2%)	Vaginal Delivery	0 (0%)
Cardiac Arrhythmias	3 (10.3%)	Caesarean section	29 (80.56%)
Valvular Insufficiency	1 (3.4%)	Emergency Caesarean section for obstetric reasons	19 (65.5%)
Thromboembolic Events	1 (3.4%)	Emergency Caesarean section for cardiac reasons	10 (34.5%)
**Obstetric and fetal outcomes**		Postpartum hemorrhage	0 (0%)
Total abortion	7 (19.4%)	**Neonatal outcomes**	
Spontaneous abortion	4 (11.1%)	Preterm birth	7 (24.1%)
Therapeutic abortion	3 (8.3%)	Birth weight, mean ±SD, g	3009 ± 710
Hypertensive disorders		Low birth weight (<2500 g)	5 (17.2%)
Pregnancy induced hypertension	2 (5.6%)	Small for gestational age	1 (3.4%)
Preeclampsia or HELLP syndrome^b^	0 (0%)	Low Apgar score (<7 at 5 min)	1 (3.4%)
Gestational diabetes mellitus	4 (11.1%)	Neonatal congenital heart disease	0 (0%)
Vaginal bleeding during pregnancy	0 (0%)	Neonatal death	0 (0%)

Data in n (%) unless otherwise specified.

^a^Maternal mortality up to 18 months postpartum.

^b^HELLP, haemolysis, elevated liver enzymes, low platelets.

### Maternal cardiac outcomes

In this cohort, there were no reports of maternal mortality or intrauterine fetal demise (IUFD). New-onset heart failure was found in 13.9% of the women, and 27.8% of them needed hospital admission for cardiac reasons during pregnancy.

Among the 29 pregnancies that progressed to delivery, all were by cesarean section. The overall cesarean rate among registered pregnancies was 80.6%, with 65.5% performed for obstetric indications and 34.5% for cardiac indications. No vaginal deliveries occurred in this cohort. Intraoperative cardiac complications occurred in 5 of the 29 cesarean deliveries (17.2%), including cardiac arrhythmias in 3 cases (10.3%) and valvular insufficiency in 1 case (3.4%). Pulmonary embolism was a rare event and was only seen in one patient (3.4%). They were comprised of one case of first-degree heart block, one case of tachyarrhythmia, and one case of ventricular tachycardia (VT) that needed cardioversion. Moreover, a patient who underwent surgery developed dyspnea and hemoptysis, while moderate valvular regurgitation was found in another patient.

### Obstetric and fetal outcomes

The overall abortion rate was 19.4%, which is made up of 11.1% spontaneous abortions and 8.3% therapeutic abortions. Thus, among the three cases of therapeutic abortion, one was because of hypoplastic left heart syndrome with the absence of the ascending aorta and the right ventricle and right atrium being beyond the normal size; another was due to cardiac arrhythmia; and the third was by ventricular failure and idiopathic cardiomyopathy. Hypertensive disorders were rather scarce as pregnancy-induced hypertension was identified in 5.6% of the women, and there were no preeclampsia or HELLP syndrome cases. Gestational diabetes was found in 11.1% of the women. There were no cases of vaginal bleeding during pregnancy or postpartum hemorrhage.

### Delivery outcomes

The average gestational age at delivery was 35.2 ± 2.4 weeks showing a large percentage of preterm delivery. All the deliveries were by cesarean section (80.6%), and there were no vaginal deliveries. Of the cesarean sections, 65.5% were performed for obstetric reasons and 34.5% for cardiac indications.

### Neonatal outcomes

The mean gestational age at delivery was 35.2 ± 2.4 weeks. Preterm birth (<37 weeks) occurred in 7 cases (24.1%). The mean birth weight was 3009 ± 710 grams. Low birth weight (<2500 g) was identified in 17.2% of the neonates, and 3.4% were small for gestational age. Only a single neonate (3.4%) had a low Apgar score (<7 at 5 minutes). No cases of congenital heart disease were identified in neonates following systematic cardiac screening, including physical examination, pulse oximetry, and selective echocardiography when indicated.

Table 4 Clark’s classification maternal risk stratification of cardiac and obstetric outcomes corresponding to the figure. Most of the women were rated as Class I or II, which indicates a mild or moderate cardiac lesion with a good pregnancy outcome and cesarean delivery at term. The majority of adverse cardiac events, i.e., new-onset heart failure, cardiac complications during surgery, and arrhythmias, were recorded in Class III patients, who had severe structural lesions such as valvular stenosis or cardiomyopathy with ventricular function moderately decreased. All the patients in Class IV (8.3%) were those in whom pregnancy was contraindicated because of extreme maternal risk and hence underwent therapeutic abortion. All of the patients in Class IV (8.3%) were those in whom pregnancy was contraindicated due to the extreme maternal risk and hence, underwent therapeutic abortion.

**Table 4:** Maternal cardiac classification based on Clark’s risk categories and associated clinical, cardiac, and obstetric outcomes among pregnant women with structural heart disease.

Clark Class	n (%)	Main Cardiac Diagnosis	Main Cardiac Diagnosis	Obstetric Outcome
Class I	11(30.6%)	Mild congenital lesions (mild MR or MVP)	None	Term cesarean delivery
Class II	13(36.1%)	Moderate valvular disease (e.g., moderate MS) or mild LV dysfunction	Mild dyspnea, no major event	Mostly term cesarean delivery
Class III	9(25.0%)	Severe valvular stenosis or cardiomyopathy (LVEF 30–40%)	New-onset HF (13.9%), arrhythmia (10.3%), intraoperative cardiac complications (17.2%)	Preterm cesarean delivery in most cases
Class IV	3(8.3%)	Cyanotic CHD, idiopathic cardiomyopathy with severe LV failure, or malignant arrhythmia	Pregnancy contraindicated; therapeutic abortion performed	Therapeutic abortion (no delivery)

ASD: atrial septal defect; MR: mitral regurgitation; MS: mitral stenosis; CHD: congenital heart disease; LV: left ventricle; LVEF: left ventricular ejection fraction; HF: heart failure.

## Discussion

The present study investigated pregnancy and obstetric outcomes among women with cardiac diseases in Kermanshah, Iran. Despite the high-risk nature of these conditions, no maternal or neonatal mortality was observed. These findings underscore the importance of specialized care, pre-pregnancy counseling, and multidisciplinary management in improving outcomes for women with cardiac disease.

One notable point in the current study was the high prevalence of congenital heart disease (38.9%), which differs from many international studies that have reported valvular diseases or cardiomyopathies as more prevalent [24]. This discrepancy may be due to regional differences in disease prevalence or improvements in prenatal diagnostic methods for congenital heart diseases. Additionally, the high proportion of women with known cardiac disease before pregnancy (88.9%) and receiving specialized pre-pregnancy counseling (86.2%) in this study likely played a significant role in achieving favorable outcomes. Early identification and appropriate adjustment of medications, particularly discontinuation of teratogenic drugs, are considered key factors in reducing pregnancy complications. Of course, the low prevalence of pulmonary hypertension (0%) and preserved left ventricular function (94.5% of patients had LVEF ≥40%) could also be among the reasons for the absence of maternal mortality in this study, as these factors are strongly associated with adverse pregnancy outcomes [25,26].

In the current study, although no maternal or neonatal mortality was reported, the incidence of complications was significant; such that 27.8% of patients required hospitalization due to cardiac problems, and 13.9% developed new-onset heart failure. These figures align with the results of larger studies, such as the Roos-Hesselink et al. study from 2007 to 2018 involving 5,739 pregnant women with cardiac disease, which reported an 11% heart failure rate and 0.6% overall mortality. Additionally, in the ROPAC study, congenital heart disease was the most common condition (57%), and despite the increase in high-risk pregnancies, improved care has led to reduced mortality and complications [27]. This comparison demonstrates that even in resource-limited centers, the implementation of structured and team-based care can significantly reduce risks [28,29].

The high cesarean rate in this study aligns with trends observed in other populations with cardiac disease [30]. The preference for cesarean delivery in these patients is often due to concerns about hemodynamic complications during vaginal delivery. However, it should be noted that cesarean delivery can also be associated with specific complications, and decision-making should be based on the clinical condition of each patient.

Regarding fetal outcomes, the preterm birth rate (24.1%) and low birth weight rate (17.2%) in our study were lower than some studies in resource-limited countries [31] and higher than healthy populations [32]. This finding emphasizes the necessity of meticulous fetal monitoring and specialized prenatal care. The absence of congenital cardiac anomalies in neonates could also be attributed to the type of predominant diseases in the mothers (e.g., atrial septal defects with low heritability). Meanwhile, in a prospective study aimed at investigating the impact of maternal cardiac disease on fetal cardiac function, 51 pregnant women with cardiac disease (including congenital heart disease, valvular disease, cardiomyopathy, etc.) and 51 healthy pregnant women as a control group were evaluated. Fetal cardiac function in both groups was assessed using fetal echocardiography in the second and third trimesters. The results showed that fetuses of mothers with cardiac disease had higher right and left ventricular Tei indices compared to the control group, indicating relative impairment in systolic and diastolic function of the fetal heart. Overall, this study demonstrated that maternal cardiac disease can cause mild yet significant changes in fetal cardiac function and underscores the importance of meticulous fetal cardiac monitoring in these mothers [33]. In the current study, fetal cardiac function was not systematically evaluated during pregnancy. Although no congenital heart defects were detected in neonates following systematic postnatal screening, this may reflect the small sample size and the types of maternal cardiac lesions (e.g., atrial septal defects with low heritability) rather than a true absence of risk. Future research with larger cohorts should consider incorporating fetal echocardiographic surveillance to better characterize fetal cardiac outcomes in this population.

There are limitations in this study that warrant being taken into account before the results are interpreted. One of such limitations is the relatively small sample size (only 36 patients) that made the statistical power of the analyses very low and it was quite difficult to find the subtle associations. The dependence on a single medical center only may cause selection bias and the results may not be applicable to other communities and medical centers. The restriction on undiagnosed pre-pregnancy cardiac conditions and the relatively short follow-up period (18 months) are also some of the limitations that have to be considered. Besides that, even though low income and limited education as a part of the socio-economic background of the study population were quite pronounced, the effect of these factors on the results was poorly analyzed, thereby, opening up the possibility of understanding health care inequality better and how it influences the health of this group of pregnant women.

## Conclusion

This prospective registry-based study characterized the clinical features, risk factors, and pregnancy outcomes of 36 women with structural heart disease in Kermanshah, Iran. The findings demonstrate that organized pre-pregnancy counseling and multidisciplinary care were associated with zero maternal and neonatal mortality, despite a 24.1% preterm birth rate and 27.8% rate of cardiac hospitalization. These results highlight the value of registry-based approaches in documenting regional patterns of cardiac disease during pregnancy and identifying areas for targeted intervention. Expansion of such registries and development of standardized care protocols are warranted to further improve maternal and neonatal outcomes in this high-risk population.

## Supporting information

S1 Dataset Supplementary data. (XLSX)

S2 Dataset Supplementary data. (XLSX)
